# Public awareness of three major infectious diseases in rural Zhejiang province, China: a cross-sectional study

**DOI:** 10.1186/1471-2334-13-192

**Published:** 2013-04-29

**Authors:** He Liu, Mei Li, Mingjuan Jin, Fangyuan Jing, Hui Wang, Kun Chen

**Affiliations:** 1 Department of Epidemiology and Health Statistics, School of Public Health, Zhejiang University, Hangzhou, Zhejiang, China

**Keywords:** Awareness, Prevention, Infectious disease, Rural residents

## Abstract

**Background:**

This study aimed to investigate the level of awareness of and factors associated with major infectious diseases in rural China and to provide the most recent baseline data for the prevention and control of these diseases.

**Methods:**

This cross-sectional study was carried out in Zhejiang province between December 2010 and April 2011. Participants were recruited from 36 villages and interviewed by doctors from the community health service using a structured questionnaire.

**Results:**

The study sample consisted of 36,377 subjects aged 15 to 80 years old. Study results showed that knowledge of HIV was adequate in 44.21% of rural residents; knowledge of TB was adequate in 52.66% of respondents; and knowledge of HBV was adequate in 60.18% of respondents. People in older age groups and with lower education levels were more likely to have low levels of awareness of these three infectious diseases. Participants in the farming industry had poorer awareness of HIV and HBV, while students and factory workers knew little of TB. The proportions of people reporting being fully satisfied with the control policies for HIV, TB and HBV were 37.70%, 34.25% and 36.12%, respectively.

**Conclusions:**

The level of awareness of HIV, TB and HBV is still low among rural residents. Further national disease control plans for major infectious diseases should emphasise effective and comprehensive health education campaigns to increase public awareness of these diseases in rural areas of China.

## Background

Infectious with the human immunodeficiency virus (HIV), tuberculosis (TB) and hepatitis B virus (HBV) infection are major public health problems in many parts of the world. In China, approximately 780,000 people live with HIV; 26,000 died from Acquired Immune Deficiency Syndrome (AIDS) in 2009 [1], while 4.5 million Chinese have been infected with active pulmonary TB, 1.5 million of whom are smear-positive [2]; meanwhile 120 million people have been chronically infected with HBV [3], and more than 300,000 people die from HBV-related diseases every year [4]. According to the 6^th^ national population census, over 0.67 billion people live in rural areas and account for 50.32% of the entire population. Rural residents differ from urban residents in terms of socioeconomic status, which can lead to heightened infectious diseases risks [2,5].

Public awareness of infectious diseases plays an important role in disease control; a lack of reasonable knowledge of infectious diseases leads to low detection rates, the interruption of treatment, discrimination and stigma. Therefore, to stop the spread of HIV, TB and HBV in China, the Chinese ministry of health launched specific national disease control plans [6-8], using posters, advertisements on television and printed media and other methods to improve the awareness of these diseases in the general population. The assessment of the awareness levels in rural residents is very important because it helps determine the impact of previous prevention efforts made by the government and gauge the need for interventions.

Although several researchers have investigated the awareness of HIV, TB and HBV of rural Chinese inhabitants, these studies have often focused on high-risk groups, such as men who have sex with men, pregnant women, or healthcare and public health professionals [9-12], and involve only one infectious disease. Very little research exists that sheds light on knowledge of and beliefs related to infectious diseases among the general rural population in China. A study [9] examining TB knowledge in a national survey conducted in China revealed that 89% of all respondents were aware of TB; the proportions were 87.3% and 95.5% in rural and urban areas, respectively. In addition, HIV infection is an important risk factor associated with active TB disease and the natural progression of liver disease caused by infection with HBV [13,14]. It is necessary to investigate the knowledge of these three infectious diseases together for their possible connection.

Zhejiang Province is one of the most densely populated provinces in China, with approximately 33.5 million urban residents and 20.9 million rural residents. In this study, we conducted a population-based epidemiological study to assess current levels of awareness of HIV, TB and HBV among rural residents in Zhejiang Province and to explore sociodemographic determinants of the awareness of these three infectious diseases.

## Methods

### Population and sampling

This cross-sectional study was conducted between December 2010 and April 2011 in rural areas of Zhejiang province in the south of the Yangtze River Delta on the southeast coast of China. Residents aged 15 to 80 years old who had lived in their residences more than 6 months before the survey were recruited into this study. The study was approved by the Ethics Committee of Zhejiang University.

The study population was obtained from the lists of residents using multistage cluster sampling. Firstly, 12 counties from 6 cities in Zhejiang Province were randomly selected. Secondly, each county was divided into three groups according to the economic levels; one town was then randomly selected from each group. Finally, one village community was selected among the 36 towns; 1200 rural residents from the community meeting the criteria were selected as the target population. If the total number of participants (1200) could not be achieved in one village, another village from the same town would be selected to reach the goal.

### Questionnaire and data collection

The questionnaire was designed by the Department of Epidemiology and Health Statistics of Zhejiang University according to relevant literatures [9,15,16]; the questionnaire was pre-tested among 100 people who did not participate in the study and was modified as necessary. The questionnaire consisted of 36 questions in three sections. The first section included sample demographic characteristics, including age, sex, education, occupation, marital status and census registry. The second section involved items that related to participants’ awareness of the three infectious diseases (Table 1); six of the items were related to HIV knowledge, five to TB knowledge and five to HBV knowledge. Each question was awarded 1 mark (total 16 marks) for the correct answer, while each incorrect or unknown answer was given 0 marks. The total marks of questions were evaluated as follows: scores of 75% to 100% were considered “excellent”, while scores of 50% to 74%, 25% to 49% and 0% to 24% were considered “good”, “poor” and “very poor”, respectively. In analysing the relationship between awareness of the infectious diseases and demographics, “aware” was defined as “excellent” and ‘good’, while “not-aware” was defined as “poor” and “very poor”. The third section consisted of questions related to health publicity and satisfaction with control policies for these three infectious diseases.

**Table 1:** Distribution of knowledge of three infectious diseases in a rural population in Zhejiang Province, China, December 2010- April 2011 (N = 36,377)

**Items**	**n**	**%**
**HIV knowledge**		
Dinning or working with an HIV-infected person is a dangerous behaviour?		
Yes	5,665	15.57
No	22,196	61.02
Unknown	8,516	23.41
If a person looks healthy, could he or she be an HIV-carrier?		
Yes	22,360	61.47
No	3,614	9.93
Unknown	10,403	28.60
If the mother was infected with HIV, could HIV/AIDS be spread during childbirth?		
Yes	27,591	75.85
No	1,639	4.51
Unknown	7,147	19.65
Can HIV/AIDS be prevented?		
Yes	26,431	72.66
No	2,238	6.15
Unknown	7,708	21.19
Have you ever heard of voluntary counselling and testing (VCT)?		
Yes	21,609	59.40
No	14,768	40.60
Should a person living with HIV/AIDS (PLWHA) be isolated?		
Yes	10,591	29.11
No	16,863	46.36
Unknown	8,923	24.53
**TB knowledge**		
Can TB be transmitted by coughing and sneezing?		
Yes	27,620	75.93
No	3,531	9.71
Unknown	5,226	14.37
Can you describe the main symptoms of TB?		
Yes	12,549	34.50
Partly	17,050	46.87
Unknown	6,778	18.63
If you experience expectoration and coughing for more than 2 weeks, would you go to hospital to get tests?		
Yes	33,099	90.99
No	1,946	5.35
Unknown	1,332	3.66
Do you think that most of TB can be cured?		
Yes	27,799	76.42
No	2,729	7.50
Unknown	5,849	16.08
Are you aware of the TB free detection/treatment policy?		
Yes	23,598	64.87
No	12,779	35.13
**HBV knowledge**		
Do you agree that HBV infection can cause liver cancer?		
Yes	20,198	55.52
No	5,223	14.36
Unknown	10,956	30.12
Can HBV be spread during childbirth?		
Yes	26,778	73.61
No	2,747	7.55
Unknown	6,852	18.84
Have you ever shared toothbrushes with members of your family?		
Yes	6,782	18.64
No	29,595	81.36
Do you think that HBV can be prevented by vaccines?		
Yes	29,741	81.76
No	1,930	5.31
Unknown	4,706	12.94
If you are a healthy person, would you be willing to pay for hepatitis B vaccination?		
Yes	24,297	66.79
No	8,812	24.22
Unknown	3,268	8.98

Participants were interviewed face-to-face by investigators from the local community health service centre who were trained before the survey. If there was any difficulty understanding questions, the questions were asked again without any explanation. All participants provided verbal informed consent before the survey. In case of refusal to participate or subjects worked in the city permanently (seasonal workers), the subject was dropped.

### Data analysis

Data were managed and analysed using SAS 9.2 (SAS Institute Inc, Cary, North Carolina). Descriptive data were presented as mean ± standard deviation (SD) and percentages. The Pearson *χ*^2^ test and Student’s *t*-test were used for analytical evaluation. Multivariate analysis was performed with multiple logistic regression to determine significant independent variables for predicting infectious diseases awareness. All predictor variables were entered in the equation as dummy variables. The odds ratio (OR) and 95% confidence intervals (CI) of the predicting variables were also estimated using logistic regression. The alpha level of significance for the multivariate analysis was set at 0.05 for a two-tailed test (i.e., p < 0.05).

## Results

### Demographic characteristics of respondents

A total of 39,728 people participated in the investigation; 3,351 surveys were excluded because of incomplete survey responses. As a result, 36,377 residents completed the interview. Ages of the study participants ranged from 15 and 80 years and the mean age was 46.59 years (SD = 15.33). The majority (60.16%) of subjects were farmers and almost half of them (46.47%) had only an elementary school education or less. Most respondents were married (83.74%), and registered in their local rural residence (95.96%). The percentage of participants having been exposed to health publicity related to HIV, TB and HBV over the past year was 76.39%, and 77.55%, 79.12%, respectively (Table 2).

**Table 2:** Demographic characteristics of the rural population of Zhejiang Province, China, December 2010- April 2011 (N = 36,377)

**Characteristic**	**n**	**%**
Age group, years		
15~29	5,788	15.91
30~44	10,371	28.51
45~59	12,293	33.79
≥60	7,925	21.79
Sex		
Male	18,261	50.20
Female	18,116	49.80
Education		
Primary school or illiterate	16,541	46.47
Junior high school	14,218	39.09
Senior high school	4,091	11.25
Above senior high school	1,527	4.20
Occupation		
Farming	21,885	60.16
Factory worker	4,860	13.36
Student	1,668	4.59
Unemployed	3,176	8.73
Administration and management	684	1.88
Commercial/food services	1,993	5.48
Other	2,111	5.80
Marital status		
Currently married	30,462	83.74
Currently unmarried	5,915	16.26
Census registry		
Local	34,907	95.96
Non-local	1,470	4.04
Exposed to health publicity in the last year		
HIV	27,789	76.39
TB	28,113	77.55
HBV	28,780	79.12

### Knowledge of HIV, TB and HBV

The responses to questions related to knowledge of diseases are provided in Table 1. A total of 60.18% (95%CI: 59.67-60.68) and 52.66% (95%CI: 52.15-53.17) of respondents had excellent awareness of HBV and TB, respectively; less than half (44.21% (95%CI: 43.70-44.72)) of the rural residents had excellent awareness of HIV. In additions, 16.73% (95%CI: 16.35-17.11) of respondents had very poor awareness of HIV, which was significantly higher than the percentage of respondents with very poor awareness of TB and HBV. The details of these responses are presented in Table 3.

**Table 3:** Awareness of three infectious diseases in a rural population in Zhejiang province, China, December 2010- April 2011 (N = 36,377)

	**Excellent**^**a**^		**Good**^**b**^		**Poor**^**c**^		**Very poor**^**d**^	
	**n**	**%(95%CI)**	**n**	**%(95%CI)**	**n**	**%(95%CI)**	**n**	**%(95%CI)**
HIV awareness	16,081	44.21 (43.70-44.72)	6,537	17.97 (17.58-18.36)	7,674	21.10 (20.68-21.52)	6,085	16.73 (16.35-17.11)
TB awareness	19,155	52.66 (52.15-53.17)	9,122	25.08 (24.63-25.53)	4,769	13.11 (12.76-13.46)	3,331	9.16 (8.86-9.46)
HBV awareness	21,892	60.18 (59.67-60.68)	7,139	19.63 (19.22-20.04)	4,267	11.73 (11.40-12.06)	3,079	8.46 (8.17-8.75)

^a^ Excellent = cumulative point > 75; ^b^ Good = cumulative point 50 to 74; ^c^ Poor = cumulative point 25 to 49; ^d^ Very poor = cumulative point <25.

### Factors associated with knowledge of HIV, TB and HBV

Binominal regression analysis indicated that age and educational level were predictors of awareness of HIV (Table 4). Subjects older than 60 years were more likely to score low levels of HIV awareness than those aged 15 to 29 years old (OR = 0.41, 95%CI 0.37-0.46); people with educations above high school education were nearly five times more likely to have acquired HIV awareness than those with primary education or below. Occupation was also significantly associated with HIV awareness. Most notably, people engaged in administration and management had higher levels of HIV awareness (OR = 2.46) than those in the farming industry. Married and local residents were more likely to have increased levels of HIV awareness, but the effect sizes were small. The OR for respondents who had not been exposed to health publicity for HIV was 0.14 compared with those who had been exposed. The effects of sex, marital status and census registry were not statistically significant. Similarly, the level of knowledge of TB and HBV of participants could be predicted by their age, educational levels, occupation and marital status (Tables 5, 6). Students, factory workers and the unemployed had less awareness of TB than farmers.

**Table 4:** Influence of characteristics on “aware” grade for HIV in a rural population, in Zhejiang Province, China, December 2010- April 2011 (N = 36,377)

**Characteristics**	**Aware**^**a**^**, n (%)**	**Not-aware**^**b**^**, n (%)**	**Odds ratio (95% CI)**	**P value**
	**(N = 22,618)**	**(N = 13,759)**		
Age group				
15~29	4,756 (82.17)	1,032 (17.83)	1.00	
30~44	7,806 (75.27)	2,565 (24.73)	0.91 (0.82-1.01)	0.0742
45~59	6,980 (56.78)	5,313 (43.22)	0.60 (0.54-0.67)	<0.001
≥60	3,076 (38.81)	4,849 (61.19)	0.41 (0.37-0.46)	<0.001
Sex				
Male	11,606 (63.56)	6,655 (36.44)	1.00	
Female	11,012 (60.79)	7,104 (39.21)	0.97 (0.92-1.02)	0.2279
Education				
Primary school or illiterate	7,442 (44.99)	9,099 (55.01)	1.00	
Junior high school	10,332 (72.67)	3,886 (27.33)	1.71 (1.61-1.82)	<0.001
Senior high school	3,426 (83.74)	665 (16.26)	2.50 (2.25- 2.79)	<0.001
Above senior high school	1,418 (92.86)	109 (7.14)	4.97 (3.97-6.23)	<0.001
Occupation				
Farming	12,166 (55.59)	9,719 (44.41)	1.00	
Factory worker	3,431 (70.60)	1,429 (29.40)	1.08 (1.00 -1.17)	0.0599
Student	1,394 (83.57)	274 (16.43)	1.52 (1.28-1.81)	<0.001
Unemployed	1,764 (55.54)	1,412 (44.46)	0.99 (0.90-1.08)	0.7389
Administration and management	629 (91.96)	55 (8.04)	2.46 (1.81 -3.34)	<0.001
Commercial/food services	1,619 (81.23)	374 (18.77)	1.91 (1.68-2.18)	<0.001
Other	1,615 (76.50)	496 (23.50)	1.58 (1.41-1.78)	<0.001
Marital status				
Currently married	18,535 (60.85)	11,927 (39.15)	1.00	
Currently unmarried	4,083 (69.03)	1,832 (30.97)	0.91(0.83-0.99)	0.0300
Census registry				
Local	21,606 (61.90)	13,301 (38.10)	1.00	
Non-local	1,012 (68.84)	458 (31.16)	0.87 (0.76-1.00)	0.0423
HIV health publicity				
Yes	20,580 (74.06)	7,209 (25.94)	1.00	
No	2,038 (23.73)	6,550 (76.27)	0.14 (0.13-0.15)	<0.001

^a^ aware = excellent + good; ^b^ not-aware = poor + very poor.

**Table 5:** Influence of characteristics on “aware” grade for TB in a rural population in Zhejiang Province, China, December 2010- April 2011 (N = 36,377)

**Characteristics**	**Aware**^**a**^**, n (%)**	**Not-aware**^**b**^**, n (%)**	**Odds ratio (95% CI)**	**P value**
	**(N = 28,277)**	**(N = 8,100)**		
Age group				
15~29	4,875 (84.23)	913 (15.77)	1.00	
30~44	8,818 (85.03)	1,553 (14.97)	1.07 (0.95 -1.21)	0.0316
45~59	9,443 (76.82)	2,850 (23.18)	0.87 (0.77-0.99)	0.2579
≥60	5,141 (64.87)	2,784 (35.13)	0.65 (0.57-0.74)	<0.001
Sex				
Male	14,452 (79.14)	3,809 (20.86)	1.00	
Female	13,825 (76.31)	4,291 (23.69)	0.93 (0.88-0.98)	0.0122
Education				
Primary school or illiterate	11,362 (68.69)	5,179 (31.31)	1.00	
Junior high school	11,910 (83.77)	2,308 (16.23)	1.72 (1.60-1.85)	<0.001
Senior high school	3,633 (88.80)	458 (11.20)	2.55 (2.25- 2.90)	<0.001
Above senior high school	1,372 (89.85)	155 (10.15)	2.94 (2.39-3.63)	<0.001
Occupation				
Farming	16,737 (76.48)	5,148 (23.52)	1.00	
Factory worker	3,931 (80.88)	929 (19.12)	0.77 (0.71 -0.85)	<0.001
Student	1,341 (80.40)	327 (19.60)	0.69 (0.58-0.83)	<0.001
Unemployed	2,253 (70.94)	923 (29.06)	0.82 (0.75-0.90)	<0.001
Administration and management	623 (91.08)	61 (8.92)	1.36 (1.01 -1.84)	0.0459
Commercial/food services	1,699 (85.25)	294 (14.75)	1.11 (0.96-1.28)	0.1482
Other	1,693 (80.20)	418 (19.80)	0.82 (0.72-0.93)	0.0019
Marital status				
Currently married	23,697 (77.79)	6,765 (22.21)	1.00	
Currently unmarried	4,580 (77.43)	1,335 (22.57)	0.90 (0.82-0.99)	0.0232
Census registry				
Local	27,177 (77.86)	7,730 (22.14)	1.00	
Non-local	1,100 (74.83)	370 (25.17)	0.74 (0.65-0.86)	<0.001
TB health publicity				
Yes	24,465 (87.02)	3,648 (12.98)	1.00	
No	3,812 (46.13)	4,452 (53.87)	0.15 (0.14-0.15)	<0.001

^a^ aware = excellent + good; ^b^ not-aware = poor + very poor.

**Table 6:** Influence of characteristics on “aware” grade for HBV in a rural population in Zhejiang Province, China, December 2010- April 2011 (N = 36,377)

**Characteristics**	**Aware**^**a**^**, n (%)**	**Not-aware**^**b**^**, n (%)**	**Odds ratio (95% CI)**	**P value**
	**(N = 29,031)**	**(N = 7,346)**		
Age group				
15~29	5,041 (87.09)	747 (12.91)	1.00	
30~44	9,001 (86.79)	1,370 (13.21)	1.10 (0.97 -1.25)	0.1264
45~59	9,767 (79.45)	2,526 (20.55)	0.96 (0.85-1.09)	0.5431
≥60	5,222 (65.89)	2,703 (34.11)	0.70 (0.61-0.80)	<0.001
Sex				
Male	14,631 (80.12)	3,630 (19.88)	1.00	
Female	14,400 (79.49)	3,716 (20.51)	1.05 (1.00-1.12)	0.0743
Education				
Primary school or illiterate	11,747 (71.02)	4,794 (28.98)	1.00	
Junior high school	12,169 (85.59)	2,049 (14.41)	1.56 (1.46-1.68)	<0.001
Senior high school	3,694 (90.30)	397 (9.70)	2.10 (1.82- 2.37)	<0.001
Above senior high school	1,421(93.06)	106 (6.94)	2.66 (2.10-3.37)	<0.001
Occupation				
Farming	16,703 (76.32)	5,182(23.68)	1.00	
Factory worker	4,200 (86.42)	660 (13.58)	1.33 (1.21 -1.47)	<0.001
Student	1,433 (85.91)	235 (14.09)	1.37 (1.13-1.64)	0.0011
Unemployed	2,397 (75.47)	779 (24.53)	1.01 (0.92-1.12)	0.8132
Administration and management	641(93.71)	43 (6.29)	2.05 (1.45 -2.88)	<.0001
Commercial/food services	1,806 (90.62)	187 (9.38)	1.95 (1.65-2.30)	<0.001
Other	1,851 (87.68)	260 (12.32)	1.77 (1.53-2.05)	<0.001
Marital status				
Currently married	24,335 (79.89)	6,127 (20.11)	1.00	
Currently unmarried	4,696 (79.39)	1,219 (20.61)	0.80(0.73-0.88)	<0.001
Census registry				
Local	27,802 (79.65)	7,105 (20.35)	1.00	
Non-local	1,229 (83.61)	241 (16.39)	0.94 (0.81-1.10)	0.4665
HBV health publicity				
Yes	25,042 (87.01)	3,738 (12.99)	1.00	
No	3,989 (52.51)	3,608 (47.49)	0.20 (0.18-0.21)	<0.001

^a^ aware = excellent + good; ^b^ not-aware = poor + very poor.

### Evaluation of the satisfaction of participants with control policies and sources of health publicity

In this study, a graded system was used to assess the satisfaction of respondents with control policies for the three infectious diseases; possible answers ranged from “fully satisfied” to “do not care”. Over one third of the participants (37.70%) were fully satisfied with the policy for HIV, 36.12% were fully satisfied with the HBV control policy and 34.25% were completely satisfied with the TB control policy; 3.34% of participants were dissatisfied with the HBV control policy, and lower proportions of respondents were dissatisfied with the TB and HIV policies.

The main sources of health publicity for the three infectious diseases were television (48.76%), newspaper and magazine (17.76%), broadcasts (7.27%) and relatives (7.17%). The question, “Are you satisfied with the current health publicity of these three infectious diseases?” was answered according to the following proportions: “fully satisfied (27.24%)”, “satisfied (56.52%)”, “dissatisfied (2.41%)”, “cannot decide (8.78%)”, “do not care (5.06%)”. The main reasons for dissatisfaction with health publicity were “the frequency of health education is low” (63.28%) and “content is too simple” (36.39%).

## Discussion

This study was a population-based cross-sectional study, utilising a representative sample design and based on face-to-face interviews; the goal of the study was to assess whether rural residents had knowledge of HIV, TB, and HBV.

Raising the level of knowledge of infectious diseases could not only help the general population protect themselves, but also promote those suspected suffers of being infected to seek medical help early and treat more completely [9]. In this study, respondents were more aware of HBV than the other two infectious diseases, and less than half of the respondents scored satisfactory levels of awareness of HIV. In China, the prevalence of infection with HBV is over 20 times higher than that of HIV and TB, and HBV infection causes more deaths every year than the other two diseases combined [12]. Therefore, significant differences in prevalence and mortality partly contribute to the unbalanced distribution of awareness of these three infectious diseases.

Many studies have been conducted to measure awareness levels of these three infectious diseases among the general public. One study conducted in seven provinces of China [17] found that 43% of participants had full and accurate knowledge of HIV; a similar proportion was found in the present study (44.21%). In another study conducted in Hyderabadi in the south of India [18], knowledge of HIV was higher than in this study (80.63% vs. 62.18%). Infection with HIV is strongly stigmatised in China [17,19]; increasing full and accurate knowledge of the disease is one of the most important ways of reducing negative attitudes toward person living with HIV/AIDS (PLWHA). In this study, less than half of the participants (46.36%) made it clear that PLWHA should not be isolated; this proportion is lower than that found by Lau et al. [20], possibly because our subjects were all rural residents. In Li’s study, there were still 12.3% of doctors and nurses expressed opinions that PLWHA should be isolated [21]. However, in Karnataka, nearly four-fifths of the respondents were of the opinion that PLWHA should not be isolated from society [22]. It is necessary to provide health education programmes with information on the mode of transmission of the disease and to develop a series of policies and measures to fight against HIV stigmatisation and discrimination in China [23]. Awareness alone is not sufficient for eliminating stigmatising attitudes and behaviour. The completeness and equity of relevant laws to PLWHA are also necessary for decreasing discrimination against PLWHA.

Partially because of the recent progress in TB prevention in China, most questions related to TB knowledge in our survey were correctly answered by more than half of the participants. Three-quarters of the participants (76.42%) knew that TB is curable in our study, while only 58.5% of the respondents in a large national survey [9] and 32% of respondents in a US National Health Interview Survey (2000–2005) [24] were aware of this fact. Nevertheless, we found that only one-third of rural participants were aware of the main TB symptom; this lack of knowledge could delay or prevent suspected infected people from seeking professional help.

The rural residents also fell short when it came to knowledge of HBV infection, and only half of the participants were accurately aware that HBV infection can cause liver cancer; 73.61% of participants recognised that HBV could be congenitally transmitted, a proportion similar to that found in a study of Chinese immigrants [25,26]. In other published studies [12,15] conducted among Chinese sub-groups, approximately one-third (29%) of high trained Chinese healthcare and public health professionals did not know that the chronic HBV infection confers a high risk of liver cancer, and 34% of respondents failed to identify all of modes of HBV transmission; similarly, only 39.2% of Chinese and southeast Asian Canadians accurately answered the question. Such a lack of knowledge in the general population and in sub-groups in China is a result of insufficient preventive practices that enable the persistence of high rates of HBV infection.

The Chinese government has implemented universal hepatitis B vaccination as part of their national infant immunisation program since the 1990s to reduce the hepatitis B burden in the overall population of China [27,28]. Since then, the prevalence of hepatitis B in China has apparently declined, especially in children, though the prevalence in adults is still high. Based on our findings, 81.76% of rural residents thought that hepatitis B can be prevented by vaccines; however, only 66.79% of participants would be willing to pay for such a vaccine. Consequently, nearly 15% of rural residents would not pay for the hepatitis B vaccine even with knowledge of its effective protective ability. The main reason for this might be the lack of individual economic means [29] and low levels of awareness of HBV. Therefore, free hepatitis B vaccination should be applied to the adults of rural areas to reduce the heavy burden of health systems.

Age and education were significantly associated with awareness of these three infectious diseases. Older respondents and those with a low educational level had lower levels of knowledge of all aspects of these three infectious diseases. In addition, farmers and the unemployed tended to have low level of knowledge of HIV and HBV. Students and factory workers were more likely to have low levels of TB awareness than farmers. We were gratified to find that nearly three-quarters of rural residents have been exposed to health publicity related to these three infectious diseases in the last year and we were encouraged to see that those who received health publicity tended to have higher levels of awareness of these diseases. The main sources of health education were television, newspaper and magazine, which may be difficult for the elderly and minimally educated subjects to understand. It is clear that there is a need for more multifaceted, comprehensive health publicity campaigns in schools, factories, and community health services to successfully increase the levels of awareness of these infectious diseases among rural residents. In rural areas of China, old age and farming have always been associated with low levels of education. Therefore, education should always be a national long-term strategy [9].

To control these three main infectious diseases, the government has made efforts to provide care and support to infected people and to prevent the occurrence of these diseases among the general population. From the first “Medium-and Long-Term Programs for the Prevention and Control of AIDS” in 1997 to the “Four Frees and One Care” program in 2004 [30], the government has prioritised interventions to control this epidemic in high risk populations, implemented routine HIV testing in population and provided free treatment of infected individuals [31]. Meanwhile, the Directly Observed Treatment, Short-course (DOTS) strategy [32] and a free hepatitis B vaccine program recommended by WHO for all neonates [12] were implemented in China. These policies in China have had significant impacts on the enhancement of disease control. In our study, approximately one third of rural residents were fully satisfied with the policies. The HIV policy was found to be highly satisfactory to the highest number of people, while the TB policy was found to be the least satisfactory. However, the majority of rural residents were dissatisfied with the HBV policy, possibly because the Chinese governments have supplied free treatment for HIV and TB but not for HBV infection. As a life-long disease, the cost of treating HBV infection can only be partly reimbursed by rural cooperative medical insurance; it therefore constitutes a heavy burden for the majority of rural patients. The gap between national policies and actual practices, especially in HBV infection, remains a problem.

One of the limitations of this study was the selection bias due to the unavailability of seasonal workers who worked away from their village of residence. Rural population flow has always been a common social phenomenon in Chinese society and seasonal workers are always at high risk of major infectious diseases. Secondly, other factors such as financial situation and family history that could have affected the findings of this study were excluded; these factors should be the subject of further research.

## Conclusions

This population-based study demonstrated that the present level of awareness of major infectious diseases in rural residents in China is still very low; further effective health education campaigns for major infectious diseases are urgently needed. In addition, older and less educated individuals and farmers were less knowledgeable about these diseases. Prevention programs should target these individuals and provide more acceptable patterns of publicity for them. This study might also be useful for planning policies for the prevention and control of major infectious diseases. Future national disease control plans in rural areas may consider using the results of the present study as a baseline for assessing the effectiveness of the disease campaigns.

## Competing interests

The authors declare that they have no competing interests.

## Authors’ contributions

HL and KC conceived the idea, statistical analysis and wrote the manuscript. MJ and ML participated in data analysis and helped to draft and revise the manuscript critically. FJ and HW, HL participated in the field survey and design of the questionnaire, data gathering. All authors read and approved the final manuscript.

## Pre-publication history

The pre-publication history for this paper can be accessed here:

http://www.biomedcentral.com/1471-2334/13/192/prepub
